# Ultrastructural analysis of development of myocardium in calreticulin-deficient mice

**DOI:** 10.1186/1471-213X-6-54

**Published:** 2006-11-19

**Authors:** Mira D Lozyk, Sylvia Papp, Xiaochu Zhang, Kimitoshi Nakamura, Marek Michalak, Michal Opas

**Affiliations:** 1 Department of Laboratory Medicine and Pathobiology, University of Toronto, Toronto, Ontario, M5S 1A8, Canada; 2 Kumamoto University School of Medicine, Department of Pediatrics, Kumamoto, Japan; 3 Canadian Institutes of Health Research Group in Molecular Biology of Membranes, Department of Biochemistry, University of Alberta, Edmonton, Alberta, Canada

## Abstract

**Background:**

Calreticulin is a Ca^2+ ^binding chaperone of the endoplasmic reticulum which influences gene expression and cell adhesion. The levels of both vinculin and N-cadherin are induced by calreticulin expression, which play important roles in cell adhesiveness. Cardiac development is strictly dependent upon the ability of cells to adhere to their substratum and to communicate with their neighbours.

**Results:**

We show here that the levels of N-cadherin are downregulated in calreticulin-deficient mouse embryonic hearts, which may lead to the disarray and wavy appearance of myofibrils in these mice, which we detected at all investigated stages of cardiac development. Calreticulin wild type mice exhibited straight, thick and abundant myofibrils, which were in stark contrast to the thin, less numerous, disorganized myofibrils of the calreticulin-deficient hearts. Interestingly, these major differences were only detected in the developing ventricles while the atria of both calreticulin phenotypes were similar in appearance at all developmental stages. Glycogen also accumulated in the ventricles of calreticulin-deficient mice, indicating an abnormality in cardiomyocyte metabolism.

**Conclusion:**

Calreticulin is temporarily expressed during heart development where it is required for proper myofibrillogenesis. We postulate that calreticulin be considered as a novel cardiac fetal gene.

## Background

Calreticulin is a ubiquitous calcium-binding protein with wide tissue distribution found in all eukaryotic cells with the exception of yeast [1], whose remarkable conservation implies an important biological function. In the lumen of the endoplasmic reticulum (ER), calreticulin functions as a calcium buffer and a lectin-like molecular chaperone [2], and it also modulates cell adhesiveness by regulating the expression of several genes encoding adhesion proteins, namely vinculin – a cytoskeletal protein and N-cadherin, a cell membrane protein [3-6]. Although calreticulin was originally discovered in striated muscle [7-9], its expression there is very low and no clear role has been attributed to calreticulin in striated muscle thus far [10]. Interestingly, ablation of calreticulin by homologous recombination is embryonic lethal due to faulty cardiac organogenesis [11]. Although viable calreticulin knockout (KO) embryos were obtained as late as 18.5 days *post coitum *(dpc), the majority of embryos died between 12.5 and 14.5 dpc [11,12]. Light microscopical analysis of KO embryos revealed a marked decrease in ventricular wall thickness, deep intertrabecular recesses, and increased fenestration in the ventricular walls. No significant histological changes in the atrial wall were observed at the light microscope level [11]. These findings were unexpected as calreticulin abundance in adult cardiac tissue is very low [9]. To determine the activation of the calreticulin promoter, transgenic mice expressing a Green Fluorescent Protein (GFP) reporter gene under control of the calreticulin promoter were previously generated by us, and we have shown that the activation of the calreticulin promoter occurred in a highly restricted spatiotemporal pattern during development [11]. The most intense GFP fluorescence occurred in the cardiovascular system at day 9.5 of embryonic development. The highest expression of calreticulin in embryonic hearts was observed at 13.5 dpc [11]. In older embryos, the high activity of the calreticulin promoter was maintained in the heart and arteries, but it decreased progressively by day 18.5 of embryonic development. Finally, a negligible level of fluorescence was detected in the heart of three-week old transgenic mice [11]. These findings showed that the activity of the calreticulin promoter is down regulated at late stages of development and after birth, which are in agreement with earlier observations that mature hearts express a relatively low level of calreticulin [13]. Collectively, these observations suggest that calreticulin plays an important, albeit unknown, role in cardiac development and function. The present study was undertaken to unravel the ultrastructural effects of the absence of calreticulin on cardiac tissue morphogenesis using Transmission Electron Microscopy (TEM).

## Results

The following convention has been assumed in this manuscript regarding the description of the stages of heart development: early stage of embryonic heart development refers to 12.5 to 13.5 dpc; mid-stage refers to 14.5 to 15.5 dpc; and late stage refers to 16.5 to 18.5 dpc.

### Expression of cardiac calreticulin

**Figure 1:**
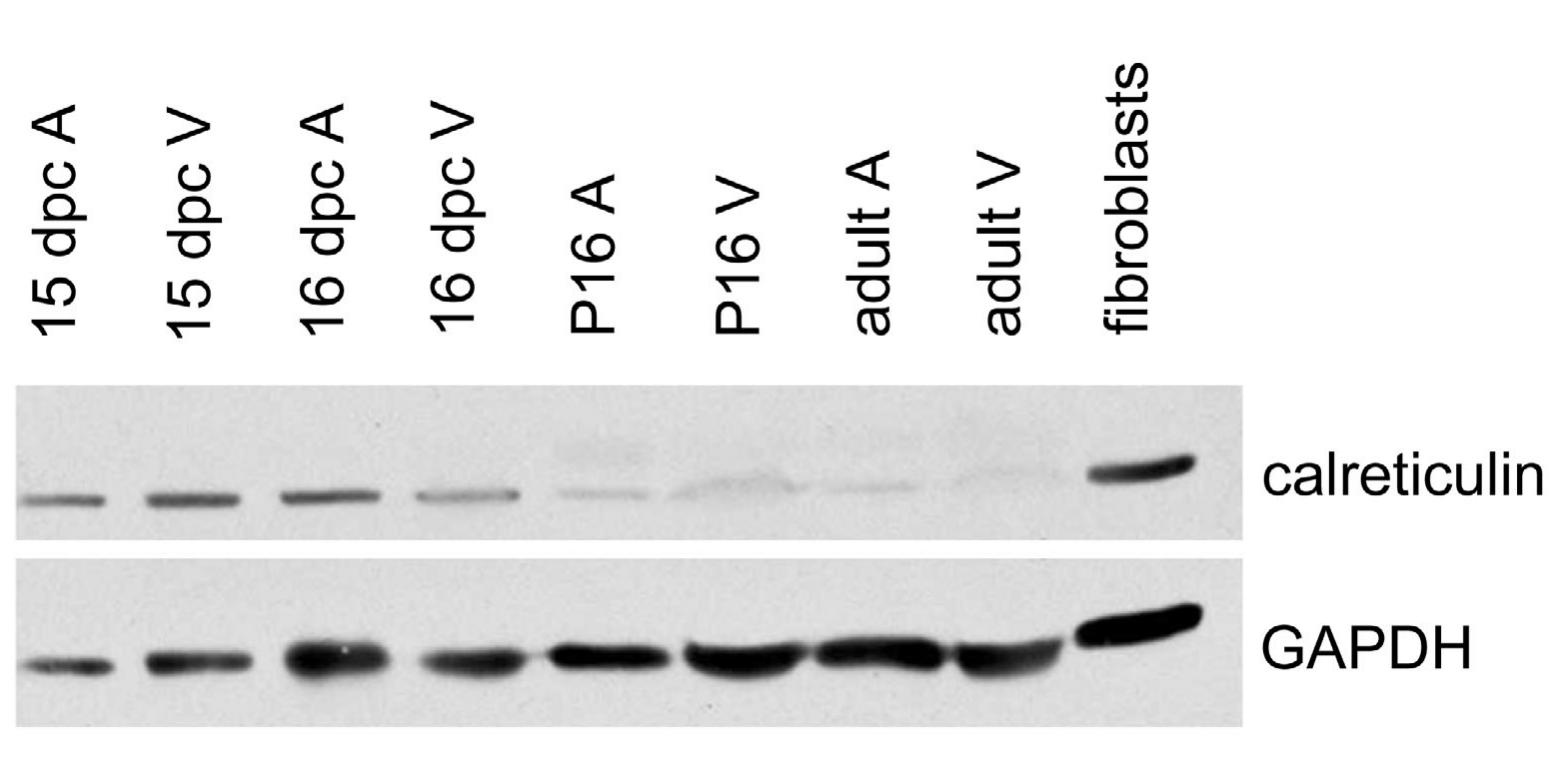
Western blot analysis of calreticulin expression during development of the heart. Calreticulin is abundant in both atria (A) and ventricles (V) during embryonic development days post coitus (dpc). Calreticulin protein levels becomes barely detectable postnatally (P) and even more so in the adult heart. GAPDH serves as a loading control.

Western blotting of wild type (WT) hearts reveals that calreticulin expression is regulated during embryonic development (Fig. 1). Calreticulin is fairly abundant in both embryonic atria and ventricles at 15–16 dpc, however, it becomes downregulated and barely detectable in postnatal (P) and adult hearts.

### Gross anatomy of the developing calreticulin KO mouse heart

Although the mouse heart is well developed by 12.5 dpc, under the light microscope the trabeculae carnae and papillary muscles are difficult to recognize. It is not until 16.5 dpc that the developing heart achieves its full prenatal configuration. While this applies to both calreticulin phenotypes, calreticulin KO embryonic hearts are smaller than WT and their ventricular walls are noticeably thinner (not shown). Furthermore, there is a discernible increase in the degree of trabeculation in the WT by 16.5 dpc, which is not observed in calreticulin KO hearts, and which explains the deep intertrabecular recesses previously found in KO mouse hearts [11]. Such gross anatomical differences between calreticulin KO and WT were not detected in atria at any stage of embryonic development investigated.

### Myocardial ultrastructure of the calreticulin KO mouse heart during development

#### Myofibrillar disarray

**Figure 2:**
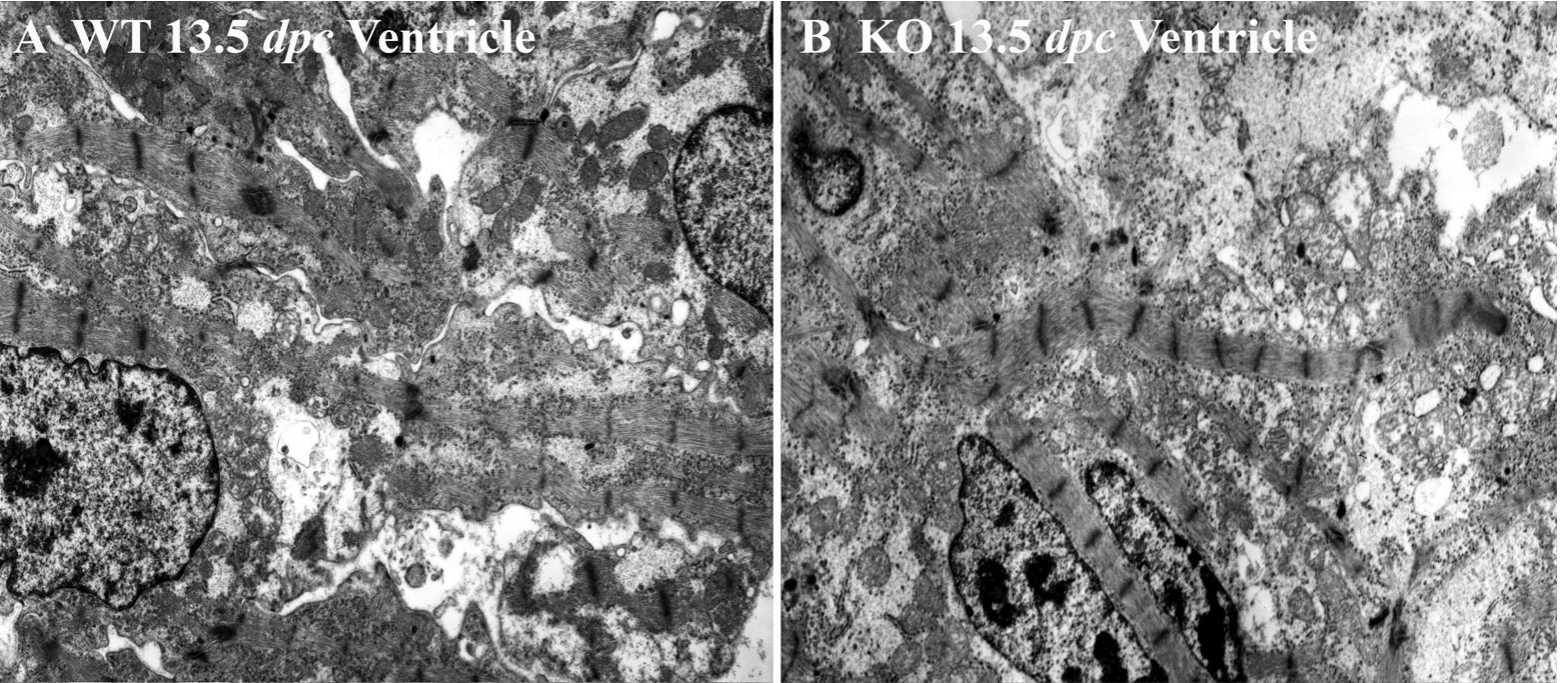
A medium power overview of myofibrillar ultrastructure in the ventricular myocardium of WT (A) and calreticulin KO (B) phenotypes. Magnification 5,000×.

**Figure 3:**
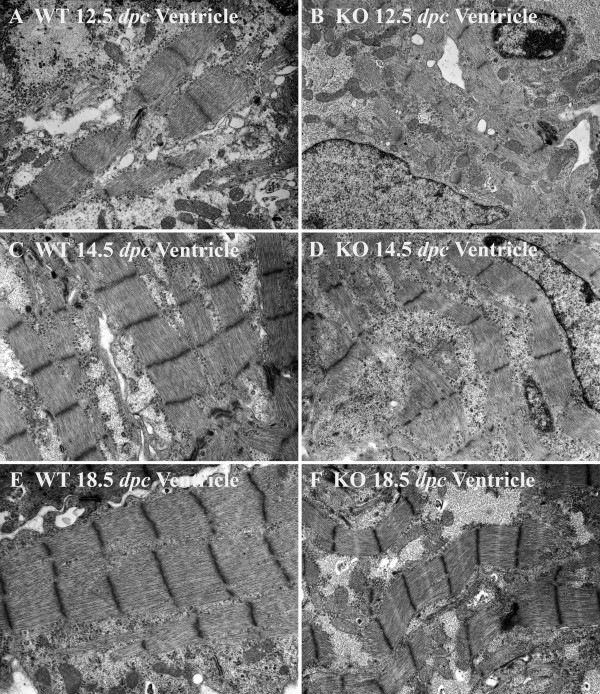
Myofibrillar ultrastructure in the developing ventricular myocardium of WT (A, C, E) and calreticulin KO hearts (B, D, F). Magnification 10,000×. At 12.5 dpc, the ventricular myofibrils of both phenotypes are only a few sarcomeres in length (A and B). These early myofibrils exhibit disarray, which is considerably more pronounced in the calreticulin KO (B) than in the WT (A) ventricular myocardium. At 14.5 dpc, ventricular myofibrils of WT (C) and to a much lesser extent the calreticulin KO (D) become less disarrayed, and start to align with the long axis of the cardiomyocyte. At 18.5 dpc, most of the ventricular myofibrils of WT phenotype (E) run in straight courses aligned parallel to each other with their Z-lines in register, thus showing little if any myofibrillar disarray. Even thought calreticulin KO myofibrils become less disarrayed with embryonic development (F), their Z-lines are frequently not aligned (F). Calreticulin KO ventricular myofibrils, already wavier in their appearance than the corresponding WT ventricular myofibrils by 13.5 dpc, do not straighten with further embryonic development as their counterparts but become visibly wavier (C vs D). This difference in the degree of waviness is most noticeable at the latest stage of embryonic development investigated, 18.5 dpc (E vs F).

**Figure 4:**
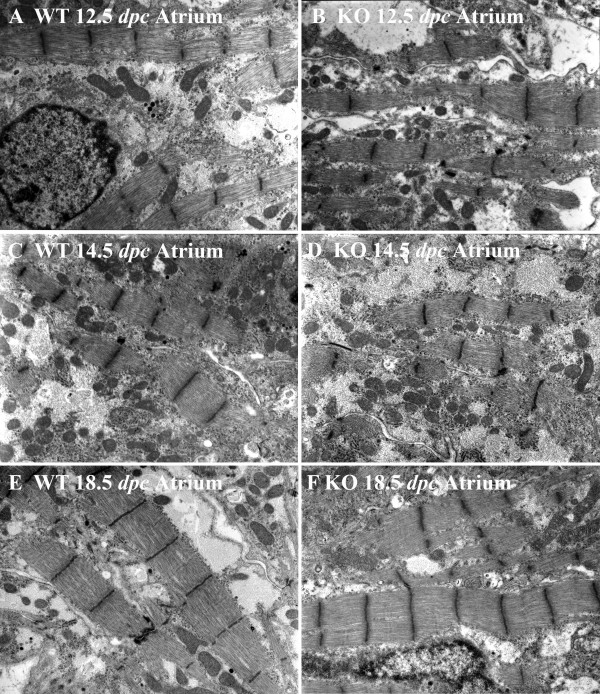
Myofibrillar ultrastructure in the developing atrial myocardium of WT (A, C, E) and calreticulin KO hearts (B, D, F). Magnification 10,000×. In atrial myocardium, there was no difference between the degree of organization following myofibrillar disarray between the calreticulin phenotypes. Atrial myocardium of both phenotypes contains large amounts of cytosol. Consequently, the myofibrillar arrangement of the atrial myocardium is less compact compared to the corresponding ventricular myocardial tissue (A, B, C, D, E, and F). By 18.5 dpc, most of the WT and KO myofibrils of atrial myocardium run in straight courses aligned parallel to each other with their Z-lines in register (E and F).

General features of ventricular myocytes in calreticulin KO and WT at an early stage of development are shown in Fig. 2. In the early stage of embryonic development, ventricular and atrial myofibrils of both calreticulin phenotypes are generally confined to the periphery of cardiomyocytes (Figs. 2A,B; 3A,B; 4A,B). During development, myofibrils increase in number and extend into the cell interior (Figs. 3C–F; 4C–F). At 12.5 dpc, these contractile elements exhibit myofibrillar disarray – a random orientation of the fibrils in the ventricles. This myofibrillar disarray is considerably more pronounced in the calreticulin KO than in the WT (Fig. 2A,B; 3A,B). In comparison, atrial myocardium is relatively unaffected (Fig. 4A,B). As development progresses, calreticulin KO and WT ventricular myofibrils become less disarrayed and begin to align parallel to the cardiomyocyte's longitudinal axis (Figs. 3C–F; 4C–F). Such increasing order in myofibrillar arrangement in WT ventricular myocardium is seen as early as 13.5 dpc, but only after 14.5 dpc in calreticulin KO (Fig. 3C,D). At 18.5 dpc, calreticulin KO ventricular myofibrils attain organization approximating that of the WT (Fig. 3E,F), although some myofibrillar disarray is still evident. In contrast to the ventricle, no delay in the myofibrillar organization is detected in the atrial myocardium (Fig. 4).

Early embryonic myocardium is characterized by the presence of large areas of electro-lucent cytosol (Figs. 2A,B; 3A,B; 4A,B). As maturation proceeds, this clear cytosolic fraction becomes reduced as the sarcoplasm becomes packed with intracellular organelles. During the period investigated, the cytosol of the atrial cardiomyocytes of both phenotypes comprises a larger portion of the sarcoplasm than that of the ventricular cardiomyocytes. Consequently, the myofibrillar arrangement of the atrial myocardium is less compact compared to the corresponding ventricular myocardial tissue (compare Figs. 3 and 4).

#### Wavy appearance of myofibrils

During the early stage of embryonic development, myofibrils are only a few sarcomeres in length and are straight in appearance (Figs. 2A,B; 3A,B; 4A,B). By 13.5–14.5 dpc, when the number of sarcomeres per myofibril increases, ventricular myofibrils acquire a wavy appearance (Fig. 3C,D; Fig. 4C,D). This waviness eventually disappears in the WT: by 18.5 dpc, the WT ventricular myofibrils run in straight courses aligned parallel to each other and with their Z-lines in register (Fig. 3E). Calreticulin KO ventricular myofibrils are much wavier at 14.5 dpc than the corresponding WT ventricular myofibrils (Fig. 3D). Furthermore, calreticulin KO ventricular myofibrils do not straighten with further embryonic development but instead become visibly wavier (Fig. 3F).

**Figure 5:**
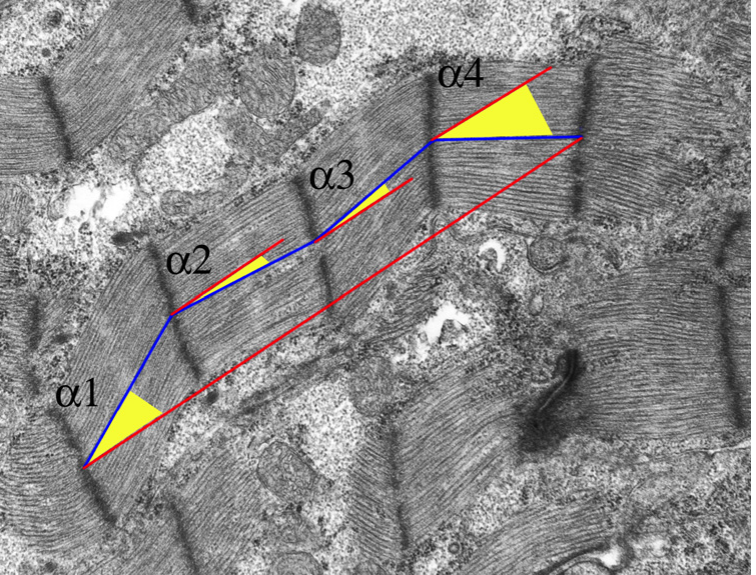
Measurement of the sarcomeric angle. An individual internal sarcomeric angle (α) was defined as an angle between a straight line connecting centres of adjacent Z-lines and a straight line connecting the centres of the initial and final Z-line of a given myofibril. To obtain average sarcomeric angle, all individual sarcomeric angles of a myofibril were averaged.

**Figure 6:**
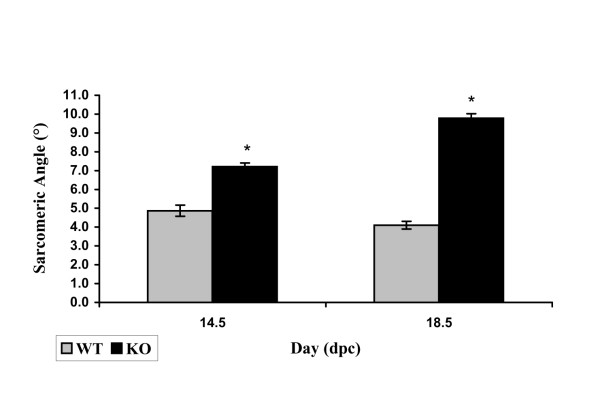
Histogram of average ventricular sarcomeric angle. Note the increase in sarcomeric angle of the KO ventricular sarcomeres compared to their WT counterparts, especially at 18.5 dpc.

To quantify the myofibrillar waviness, an angle a sarcomere makes with a trajectory between the initial and the final Z-line of the myofibril referred to as a sarcomeric angle (Fig. 5), has been adopted as a measure. Fig. 6 shows average sarcomeric angles for each calreticulin phenotype at 14.5 and 18.5 dpc. Calreticulin KO myofibrils are significantly wavier than the corresponding WT myofibrils (Wilcoxon rank sum test, P << 0.001). Percent differences in average sarcomeric angle between the WT and calreticulin KO at 14.5 and 18.5 dpc are 148% and 238%, respectively. Additional statistical analysis shows that there is a significant increase of 35% in sarcomeric angle of calreticulin KO myofibrils between 14.5 and 18.5 dpc (Wilcoxon rank sum test, P << 0.001). The myofibrillar waviness seen in calreticulin KO ventricular myocardium is not as pronounced in atrial myocardial tissue (Fig. 4C–F).

#### Myofibrillar width

**Figure 7:**
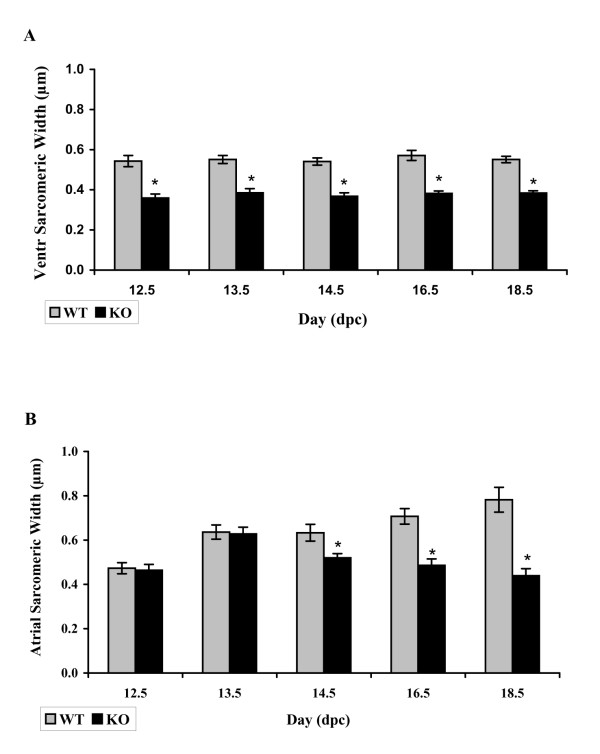
Histogram of average sarcomeric width. A: Note that the average sarcomeric width of the ventricular myofibrils is less than that of the WT myofibrils. B: Average sarcomeric width of atrial myofibrils. Commencement of thinning of the myofibrils in calreticulin KO atria in comparison to WT (taken as 100%) is evident at 14.5 dpc.

**Figure 8:**
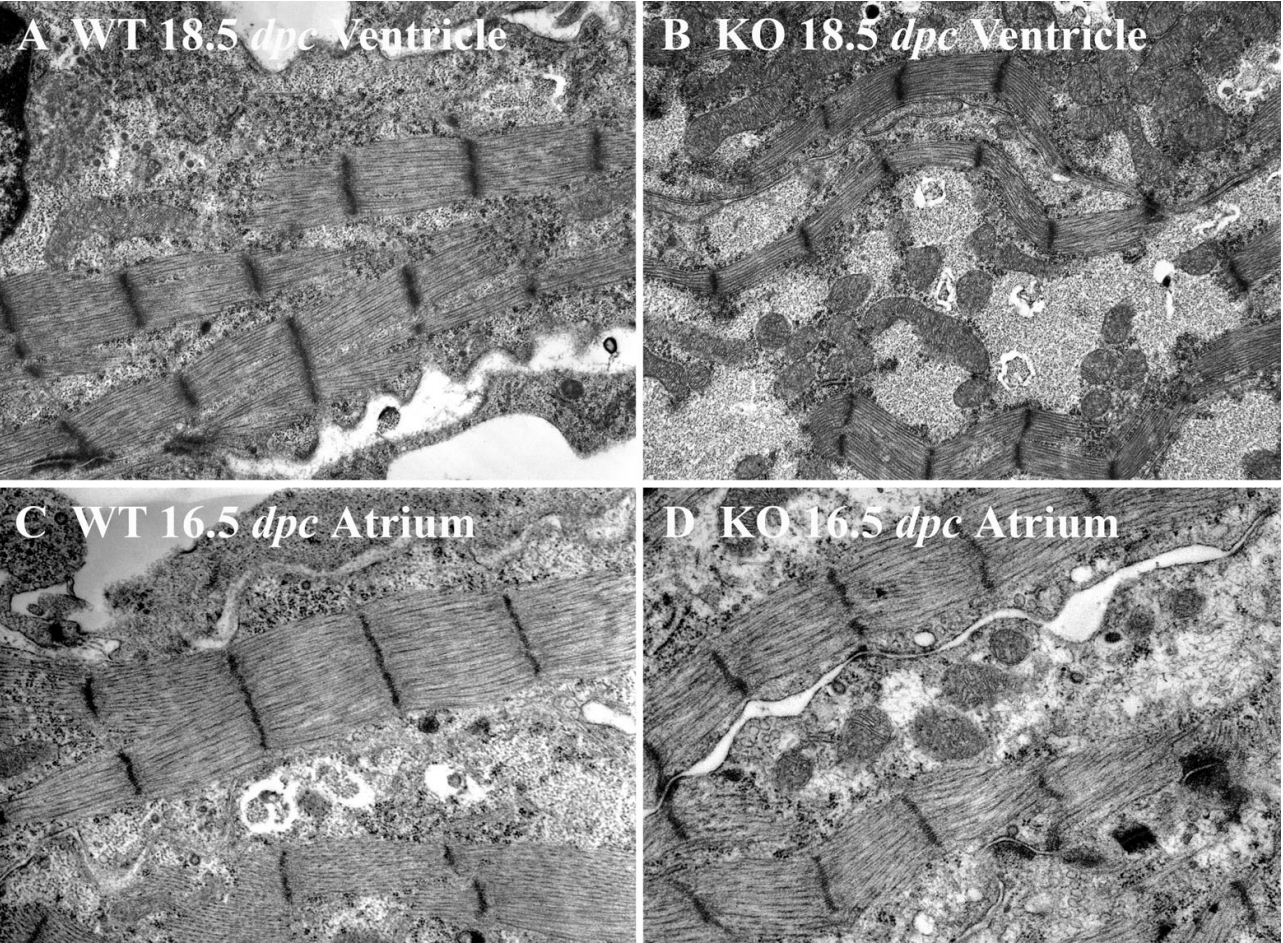
Ultrastructure of ventricular myocardium of WT (A) and calreticulin KO (B) hearts and of atrial myocardium of WT (C) and calreticulin KO (D) hearts. It is apparent that there is a substantial difference in the myofibrillar width between the WT and KO phenotypes in both ventricles and atria at late stages of development. Further details are found in the text. Magnification 15,000×.

Statistical analysis of the median ventricular sarcomeric width shows that calreticulin KO ventricular myofibrils are significantly thinner than those of the WT at all developmental ages investigated (Wilcoxon rank sum test, P < 0.05). Fig. 7A shows a histogram of average sarcomeric width of the ventricular myofibrils for each calreticulin phenotype. The calreticulin KO ventricular myofibrils are approximately 32% thinner than their WT counterparts. Figure 8 shows stark differences in ventricular myofibril width between the two phenotypes. In contrast, in the early stages of atrial development, there is no significant difference in the median sarcomeric width between the two calreticulin phenotypes (Wilcoxon rank sum test, P > 0.05). However, thinning of atrial myofibrils in the calreticulin KO heart becomes noticeable at the mid- and late stages of embryonic development (Wilcoxon rank sum test, P < 0.05) (Figs. 7B; 8).

#### Myofibrillar count

**Figure 9:**
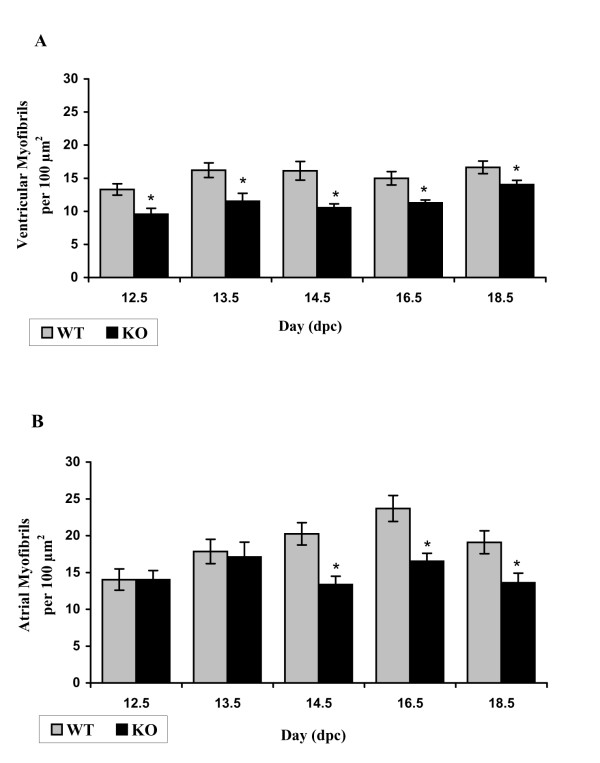
Myofibrillar count. A: Histogram of an average ventricular myofibrillar count of myofibrils for each phenotype. B: Histogram of an average atrial myofibrillar count for each phenotype.

Calreticulin KO ventricular myocardium relative to WT is characterized by a lower number of myofibrils at all stages of embryonic development investigated (Fig. 9A). In contrast to the ventricle, there is no significant difference between myofibrillar counts in the atria of calreticulin KO and WT hearts early in development. However, the myofibrillar count becomes significantly lower in calreticulin KO atria at mid- and late stages of development (t-test, P < 0.05) (Fig. 9B).

#### Z-lines

Early in embryonic development, immature ventricular and atrial Z-lines of both calreticulin phenotypes have an irregular margin and variable width (Fig. 3A,B; Fig. 4A,B). With maturation, these Z-lines narrow in width, lengthen, and become more prominent (Figs. 3C–F; 4C–F). The Z-lines of the WT ventricles and atria as well as the Z-lines of the calreticulin KO atria are often in register by mid- to late stages of embryonic development (Figs. 3C,E; 4C–F). This is in contrast to the ventricular Z-lines of the calreticulin KO phenotype, which are not well aligned, even at late stages of development (Fig. 3F).

#### Myocardial glycogen

**Figure 10:**
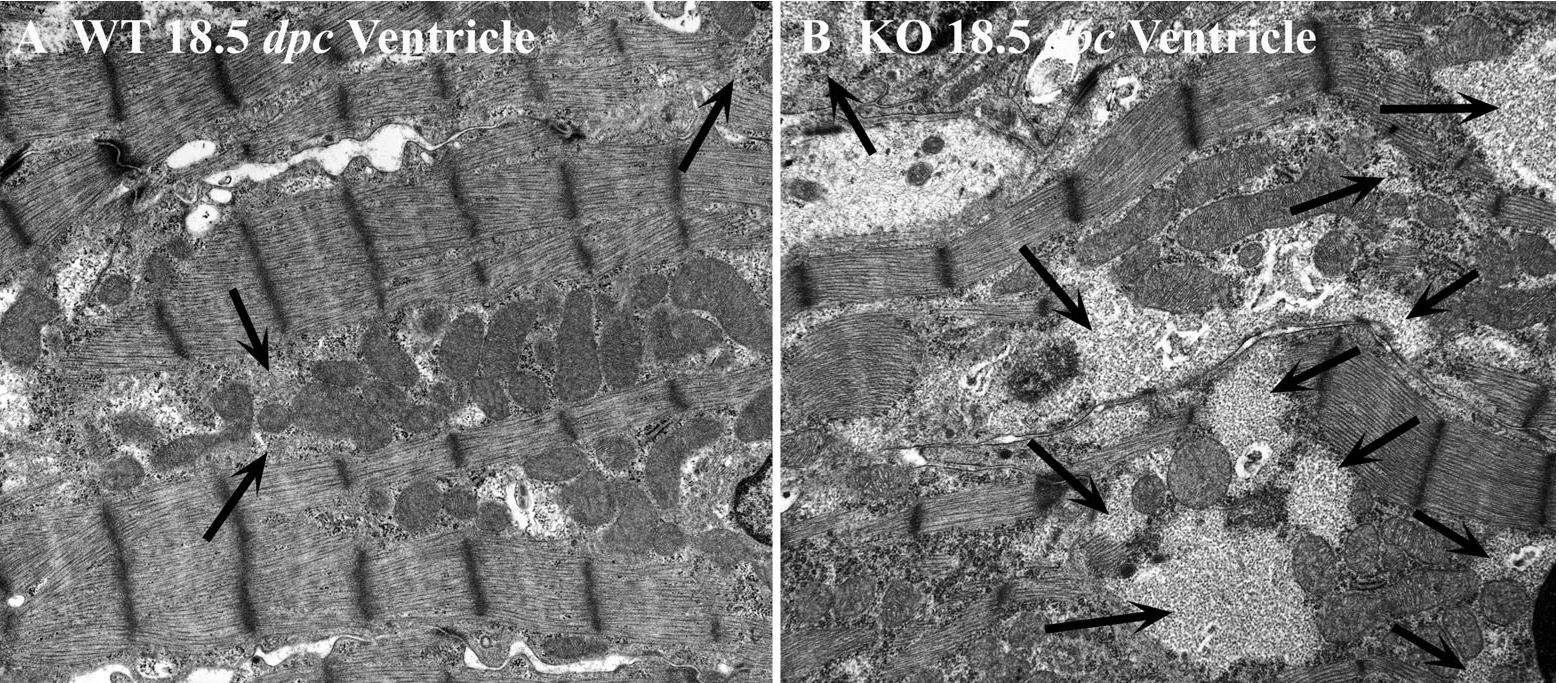
Ultrastructure of glycogen deposits in ventricular myocardium of WT (A) and calreticulin KO (B) hearts at 18.5 dpc. In calreticulin KO ventricular cardiomyocytes, glycogen is present and its abundance noticeably increases with development. Arrows point to most prominent deposits of glycogen. Further details are in the text. Magnification 12,600×.

**Figure 11:**
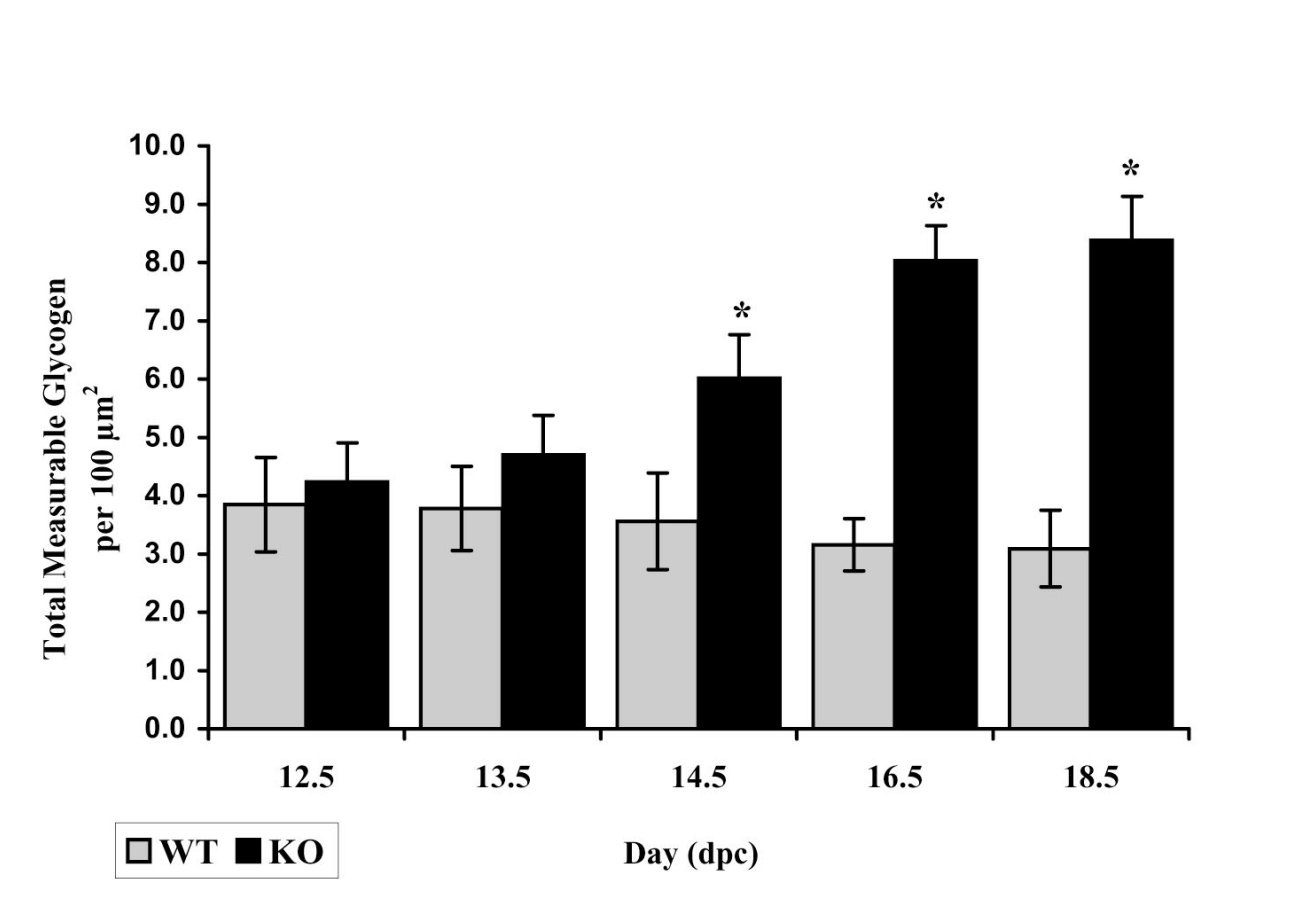
Changes in total measurable ventricular glycogen during development. Histogram of total measurable ventricular glycogen for each phenotype.

Highly branched, large molecules of glycogen appear as dark sarcoplasmic particles in electron micrographs. In the developing WT ventricular and atrial cardiomyocytes, glycogen is abundant, and its amount decreases with embryonic maturation. By 18.5 dpc, glycogen occurs mainly in large pools within the sarcoplasm, primarily concentrated in spaces between the myofibrils, and appears as large, light grey areas in electron micrographs (Fig. 10). When these glycogen pools are found near the contractile structures, they are usually located in apposition to I-bands. In contrast to WT, glycogen abundance noticeably increases with development in calreticulin KO ventricles (Fig. 10). At early stages of embryonic development, there is just slightly more glycogen in calreticulin KO ventricular sarcoplasm than in the corresponding WT sarcoplasm (Fig 11). However, as development progresses, there is a discernible and statistically significant (t-test, P < 0.05) accumulation of glycogen in the sarcoplasm of the calreticulin KO ventricular cardiomyocytes (Fig. 11). No difference in glycogen deposition was observed in the atrial myocardial tissue between the two phenotypes (data not shown).

#### Intercalated discs

**Figure 12:**
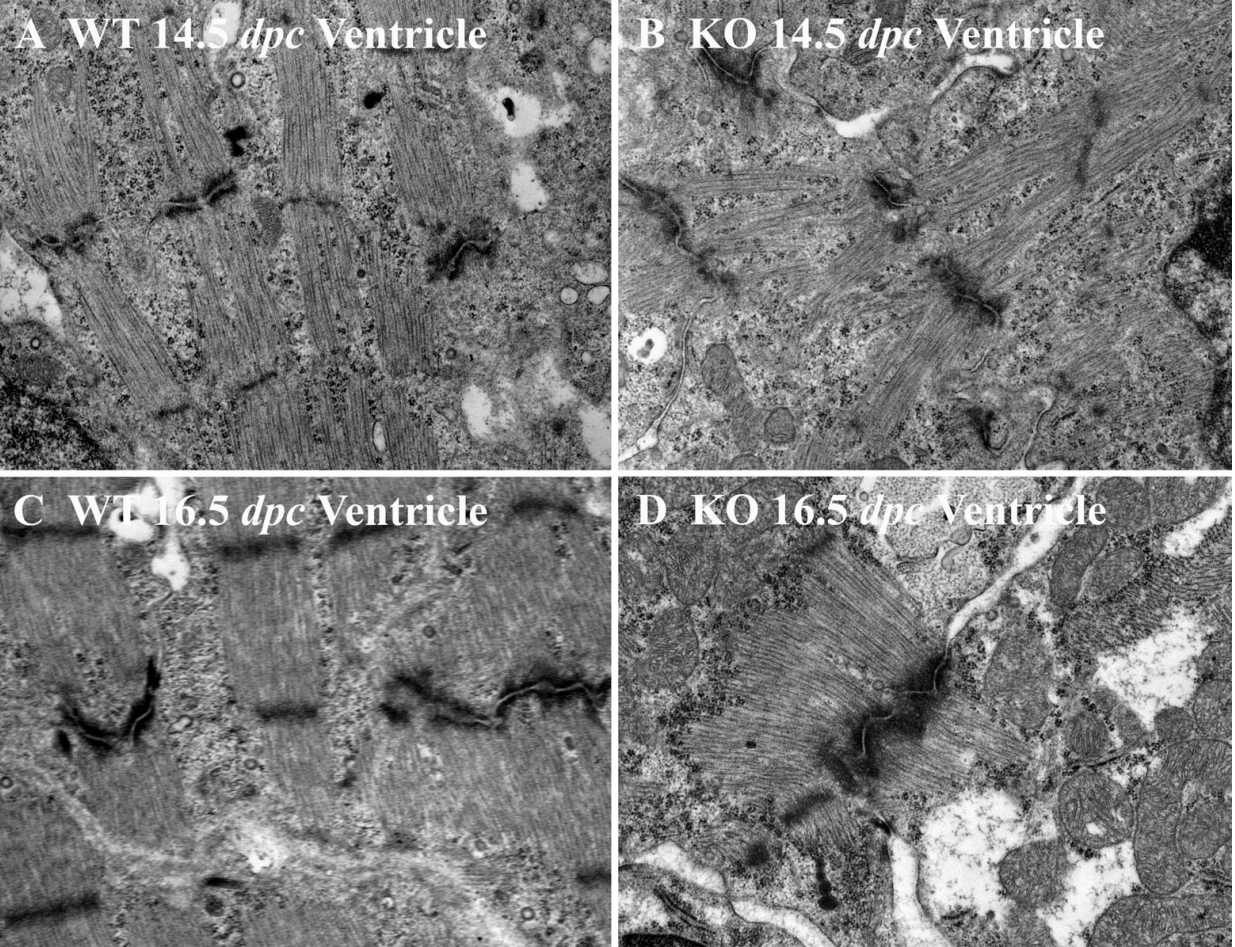
Ultrastructure of intercalated discs of the developing ventricular myocardium. Initially, the immature intercalated discs are short and thin (A, B). With embryonic development, they become larger, more complex and are accompanied by increasing inter-digitations (C, D). Additional details are in the text. Magnification 16,000×.

Early in embryonic development, immature ventricular intercalated discs of the calreticulin KO and WT mice are short, thin, and primarily oriented obliquely to the long axis of the cardiomyocyte (Fig. 12A,B). However, as development progresses, the intercalated discs become larger, more complex, and predominantly oriented perpendicularly to the cardiomyocyte axis (Fig. 12C,D). Additionally, maturation of the ventricular intercalated discs of both phenotypes is accompanied by increasing inter-digitations as seen by late stages of embryonic development (Fig. 12C,D). Quantitatively, statistical analysis reveals that at 14.5 dpc, there is no significant difference in mean ventricular number of intercalated discs between calreticulin KO and WT subject groups (Wilcoxon rank sum test: P = 0.93). Intercalated discs of the WT ventricular myocardium tend to cross cardiomyocytes in almost straight lines, but occasionally they appear in a stepwise arrangement. Interestingly, the intercalated discs of the calreticulin KO ventricular myocardial tissue are more likely to run in a stepwise fashion than the WT (not shown). No such difference was noticed in the atrial myocardium.

#### Western blot analysis of N-cadherin and vinculin expression in embryonic heart

**Figure 13:**
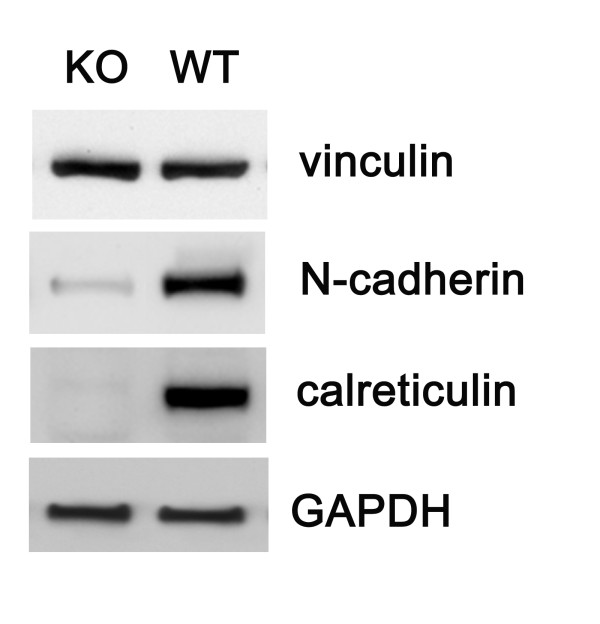
Western blot analysis of the abundance of vinculin and N cadherin in calreticulin KO and WT hearts of 14 dpc embryos. Vinculin is abundant in both phenotypes while the level of N-cadherin expression is dramatically reduced in KO hearts. Cell extract from an established line of mouse embryo fibroblasts was run in the lane marked fibroblasts, while GAPDH serves as a loading control.

Western blotting of WT and KO embryonic hearts at 14 dpc reveals that N-cadherin is substantially downregulated in calreticulin KO hearts when compared to the WT tissue (Fig. 13). Vinculin abundance does not change with ablation of calreticulin.

## Discussion

Calreticulin-null embryos die during mid-gestation due to defective heart development. Studies using mice containing GFP controlled by the calreticulin promoter showed that indeed the calreticulin promoter is highly active at the time of cardiac organogenesis but its activity decreases and becomes undetectable postnatally [11]. In this work, Western blotting revealed that the abundance of calreticulin protein follows a similar time course to that of the promoter. The novel finding presented here is that ablation of calreticulin is paralleled by myofibrillar disarrangement in the ventricular myocardium. No such changes are observed in the atrial myocardium. In addition, the calreticulin KO phenotype is characterized by a lower number of myofibrils. The major signs of myofibrillar disarrangement are random orientation, myofibrillar waviness and thinning.

Early in embryonic development, myofibrils are confined to the cardiomyocyte periphery. However, as the number and size of these myofibrils increase during development, they progressively extend into the cell interior. Myofibrils early in development exhibit myofibrillar disarray, i.e. myofibrils are randomly oriented in a section, and are not aligned parallel to each other and the long-axis of the cardiomyocyte. Such random orientation of the myofibrils is a characteristic of immature myocardium under normal conditions [17]. With the progression of embryonic development, the WT myofibrils become more aligned with one other and the long axis of the cardiomyocyte [18]. In contrast, the developing ventricular myofibrils of the calreticulin KO heart become aligned to one another and to the long axis of the cardiomyocyte much later than the ventricular myofibrils of the WT heart. This illustrates the temporary nature of this myofibrillar disorganization.

The second feature of the ventricular KO myofibrils is their wavy appearance. The waviness of WT myofibrils is most pronounced at 13.5 dpc, after which the myofibrils straighten out. Similar waviness has been described in early rat myocardium [19]. In contrast, calreticulin KO myofibrils become wavier in appearance with ongoing development instead of straightening out. This difference is most noticeable at 18.5 dpc. To verify if the myofibrillar waviness was caused by a development delay, we compared the morphology of calreticulin KO ventricular myofibrils with WT myofibrils of earlier developmental age. Such comparison revealed that the calreticulin KO ventricular myofibrils of the late stage of embryonic development do not resemble neither early nor mid-stage WT ventricular myofibrils. Hence, the wavy characteristic of the calreticulin KO ventricular myofibrils is not a result of a developmental delay but is a result of a defect associated with calreticulin deficiency.

What could be a mechanism by which calreticulin ablation could affect myofibrillar organization? Calreticulin affects the expression of both vinculin and N-cadherin in cells grown *in vitro *[3-5], which underlies its role in modulating cell adhesiveness [6]. Cell adhesion is a recognized morphogen with a role in cardiac myofibrillogenesis [20-23]. The major adhesive structures of cardiomyocytes are the intercalated discs [24,25], part of which are adherens-type junctions that contain N-cadherin [26]. N-cadherin is required for proper heart organogenesis as documented by experiments employing use of function-blocking antibodies [27-30], retroviral expression of dominant-negative mutants of N-cadherin [31,32] or genetic manipulation [33,34]. During gastrulation, N-cadherin is uniformly distributed throughout the heart forming mesoderm, and is also uniformly distributed in the tubular heart [35]. In the developing heart, zonula adherens are the primary sites of N-cadherin accumulation [26,36-38]. N-cadherin can also be found in non-junctional sarcolemma [21,27,39] and mostly in costameres [30,40]. The expression of N-cadherin in the developing myocardium, as assessed by Western blotting, gradually increases at pre-trabeculation stage, after which it remains relatively constant [32]. Importantly, the expression of N-cadherin is not as much necessary for myofibrillogenesis per se, as for continuous alignment of myofibrils between myocytes [41]. It is thus conceivable that the reduction of N-cadherin expression in calreticulin KO hearts that we report here may underlie the observed myofibrillar disarrangement.

Most myofibrillar proteins are present in the sarcomere when the fist contractions appear in the developing heart. Soon thereafter, the myofibrils acquire their finite orientation along the cell axis, probably by interaction with the intermediate filament protein, desmin, and with vinculin in the costameres, which are components of the cardiac cytoskeleton responsible for transmission of force generated by the contractile apparatus [21,23,42-46]. Thus, costameres and intercalated discs are important for proper myofibrillogenesis [17,24,40,47,48]. Comparison of the ventricular myocardium in the calreticulin KO versus WT shows very subtle differences in morphology of the intercalated discs. In WT myocardium, intercalated discs cross the cardiomyocyte in straight lines or in a stepwise fashion. Although there is no detectable morphological difference between intercalated discs in atria and ventricles of either phenotype, the intercalated discs of the calreticulin KO ventricular myocardium are more likely to run in a stepwise fashion, which is likely caused by intercalated discs of adjacent myofibrils of the cardiomyocyte not being in register. As ablation of N-cadherin causes loss of intercalated discs [34], we presume that the observed reduction in its expression may interfere with proper alignment of intercalated discs in calreticulin KO myocardium.

In developing cardiomyocytes, glycogen is abundant as it is the main energy source [49] and its amount declines with maturation. Early in embryonic development (12.5 and 13.5 dpc), glycogen is diffusely located throughout the sarcoplasm. Although glycogen in most tissues is located within lysosomes, this does not appear to be the case in the heart. Glycogen particles occur freely in the sarcoplasm: they are not surrounded by nor are attached to membranes [50,51]. By 14.5 dpc, glycogen aggregates into globules and by 18.5 dpc, it is found mainly in large pools within the sarcoplasm, which is consistent with previous reports [25]. Calreticulin KO ventricular cardiomyocytes contain significantly more glycogen than the corresponding WT cardiomyocytes. Moreover, while glycogen content decreases in WT ventricles, its amount increases with embryonic development in calreticulin KO ventricles. Developmental regulation of glycogen content in the heart has not been well studied. Knaapen et al. [52] report an increase in glycogen content in rat heart between embryonic day 11 and 17 and speculate that this increase may be more structural than metabolic. Accumulation of glycogen in calreticulin KO and not WT hearts, and in ventricles and not atria, seems to be at odds with a structural hypothesis for glycogen accumulation in the developing mouse heart. Glycogen breakdown is mediated by a cascade of events that converts inactive glycogen phosphorylase b to active phosphorylase a [49]. Calmodulin, the intracellular calcium-binding regulatory protein, is one of the subunits of phosphorylase b kinase and thus inositol trisphosphate-dependent calcium release from the stores is required for its activation and that of phosphorylase [53]. As calreticulin is the major source of inositol trisphosphate-releasable calcium [54], its deficiency may lead to impaired function of calmodulin and accumulation of glycogen. On the other hand, glycogen accumulation in ventricles of calreticulin KO resembles a mild form of glycogen storage diseases. In the glycogen storage diseases type II (Pompe's disease), type III (Cori's disease), and type IV (Andersen's disease), cardiac involvement may occur in humans [55] and mice [56]. In Pompe's disease, the cardiac muscle cells appear vacuolized by massive glycogen deposits displacing the myofibrils to the periphery of the cells [50,57,58]. N-linked glycosylation occurs during the post-translational modification of lysosomal α-glucosidase and it has been reported that mutations of lysosomal 1,4-α-glucosidase affecting its folding result in Pompe's disease [59-62]. Besides regulating intracellular calcium homeostasis, another major function of calreticulin is in "quality control" of folding of glycosylated proteins [2]. Since calreticulin functions as a lectin-like molecular chaperone, perhaps it may also be involved in the folding of the lysosomal 1,4-α-glucosidase. Thus, calreticulin deficiency might lead to improper function of the α-glycosidase and cause glycogen accumulation.

Many of the features associated with ablation of calreticulin follow a different course in the atria *versus *the ventricles. Atrial and ventricular cardiomyocytes arise from distinct cardiac myocyte lineages, and express distinct subsets of contractile proteins genes [63-65]. Thus, the absence of calreticulin may have both distinct and common effects on these cardiac phenotypes. At present, it is not clear if the defects in the calreticulin-deficient myofibrils are associated with a direct effect of calreticulin on the structure of the cardiac myofibril itself, or if calreticulin is affecting adhesive proteins, thus causing a structural change in the myofibrils. Indeed, the calreticulin KO phenotype bears similarity to other mutations ablating cell adhesion proteins [66-70], but especially to the phenotypes caused by downregulation of N-cadherin [27,29,33,41,71]. We suggest here that the morphological changes associated with the absence of calreticulin in the heart may be due to a defect in the development of the contractile apparatus and/or a defect in the development of cardiomyocyte cell adhesiveness.

## Conclusion

Calreticulin is temporarily expressed during heart development where it is required for proper myofibrillogenesis. It also appears to affect metabolism of the developing muscle via either its effects on cellular Ca^2+ ^homeostasis or its chaperone function. We thus postulate that calreticulin be considered as a novel cardiac fetal gene.

## Methods

### Animals

Calreticulin knockout mice were generated by homologous recombination as described previously by Mesaeli et al. [11]. All procedures were carried out according to the animal procedures approved by the University of Toronto.

### Dissections

Pregnant female mice at various stages of gestation were sacrificed by cervical dislocation, their uteri removed and embryos collected. Embryos were sacrificed by decapitation and the embryonic heads were used for genotyping. The embryos were then transferred into separate 3.5 cm tissue culture dishes holding fixative solution at room temperature (RT). All subsequent dissections were performed in this fixative solution. For dissection of the atria, very thin glass threads made from glass rods pulled over a Bunsen burner were used to separate the atria from the rest of the heart. The left and right ventricles were separated from each other by cutting through the ventricular septum with a razor blade.

### Sample preparation for TEM

All TEM procedures were at RT. Primary fixation was carried out for 4 hours in a freshly prepared solution of 2.5% glutaraldehyde and 2% paraformaldehyde in 0.1 M cacodylate buffer (pH 7.4) containing 1 mM of Ca^++ ^and 1 mM of Mg^++^. The samples were then washed for 1 hour in a solution of 7.5% sucrose in cacodylate buffer, followed by a second wash for 1 hour in a solution of 15% sucrose in the same buffer. Secondary fixation with 1% osmium tetroxide in 0.1 M cacodylate buffer was carried out for 1 hour. Samples were dehydrated in increasing concentrations of ethanol (30, 50, 70, 80, 90 and 100% each twice for 10 min.), followed by propylene oxide for thirty minutes. Next, samples were infiltrated with a 1:1 mixture of propylene oxide and embedding medium for 1.5 hours, followed by 100% embedding medium. Embedding in Taab 812 – Araldite 502 resin followed the formula for a block of medium hardness: Taab 812 – 12.5 g; Araldite – 7.5 g; DDSA (dodecenyl succinic anhydride) – 27 g; DMP (2, 4, 6, – tri-(dimethylaminomethyl) phenol) – 0.7 g. Overnight polymerization was carried out at 60°C. Standard thick sections of 0.6 to 0.7 μm, and thin sections of approximately 90 nm were cut with a Drukker International 2 mm diamond knife on a Reichert-Jung ultramicrotome. For optimum longitudinal orientation of the myofibrils, ventricles were cut sagittally and atria were cut transversely. Copper grids with mesh size 300, diameter 3.05 mm, were used to collect the sections. Thin sections were stained with 5% uranyl acetate in water for 15 minutes followed by lead citrate for 15 minutes. After staining, the grids were washed in double distilled water.

### TEM data collection

Heart samples were examined with a Hitachi-7000 TEM and recorded on Kodak Electron Microscope Film negatives. The areas of interest were first photographed at low magnification of 3,500× – 5,000×, then the inner myocardium was delineated, and the mesh 300 grid squares were numbered. Areas for data collection by imaging were selected randomly. A Table of Random Numbers was used to choose the area to be photographed. Data were collected from digital images of 10,000× negatives, which were scanned at the resolution of 900 pixels per inch. To determine the degree of myofibrillar wave-like appearance, sarcomeric internal angles were measured to produce an average sarcomeric angle. An individual internal sarcomeric angle was defined as an angle that a straight line connecting centres of adjacent Z-lines of a sarcomere makes with a straight line connecting the centres of the initial and final Z-line of a given myofibril (resultant) as shown in Fig. 4. All individual internal sarcomeric angles of a myofibril were then averaged to produce an average sarcomeric angle for that myofibril. Measurable glycogen area was defined as an area of at least 0.1 μm^2 ^in size. Measurements of glycogen pools were done using Adobe Photoshop. Total measurable glycogen comprises the sum of all measurable areas occupied by glycogen particles in μm^2 ^per a standard area of 100 μm^2^. Data were reported as total measurable glycogen area per phenotype in μm^2^. The Kolmogorov-Smirnov test was used to test for normality of the distribution of data between the two phenotypes. If the populations were normally distributed, as in myofibrillar count, myofibrillar thickness, and ventricular glycogen accumulation, parametric two-sample t-tests were performed to test for the difference between phenotypic groups. If the populations were not normally distributed, then the non-parametric Wilcoxon rank sum test was used.

### SDS-PAGE and western blotting

Atria and ventricles from 15.5 dpc, postnatal days (P) P1, P17 and 6-month-old mice were homogenized in ice-cold lysis buffer (50 mM Tris-HCl, 120 mM NaCl, 0.5% NP-40, pH 8.0) with protease inhibitor cocktail (Sigma), and then spun at 14,000 rpm for 15 minutes at 4°C. Samples were separated by SDS-PAGE and transferred to a nitrocellulose membrane according to a standard protocol [5,14]. All electrophoresis reagents were from Bio-Rad. The nitrocellulose membranes (0.2 μm pore size) were from Micron Separations Inc. Well-characterized goat and rabbit anti-calreticulin antibodies [7,8,15,16] were used in this study. The primary antibodies were used at the following dilutions in TBST: anti-calreticulin 1:300, anti-vinculin 1:500 (Sigma), anti-N-cadherin 1:1000 (BD Transduction Laboratories), and anti-GAPDH 1:3000 (Lab Frontier). All horseradish peroxidase conjugated secondary antibodies were used at a dilution of 1:10000 (Jackson ImmunoResearch Laboratories). Chemiluminescence ECL Plus Western blotting detection reagents were from Amersham.

## Authors' contributions

KN and MM created the calreticulin-deficient mice. MDL did the TEM work and wrote initial draft of the manuscript. SP and XZ did Western blotting. MO conceived of the study and MO and SP wrote the final manuscript. All authors read and approved the final manuscript.
